# Mucocutaneous Leishmaniasis with Rare Manifestation in the Nasal Mucosa and Cartilage Bone Septal

**DOI:** 10.1155/2020/8876020

**Published:** 2020-09-22

**Authors:** Nicole Casalle, Laís de Barros Pinto Grifoni, Ana Carolina Bosco Mendes, Sérgio Delort, Elaine Maria Sgavioli Massucato

**Affiliations:** ^1^Department of Diagnosis and Surgery, Araraquara Dental School, Universidade Estadual Paulista (UNESP), São Paulo, Araraquara, Brazil; ^2^Dermatologist Contributor on Oral Medicine Service-UNESP, São Paulo, Araraquara, Brazil

## Abstract

*Backgroud*. Leishmaniasis is an infectious disease caused by protozoan of the genus *Leishmania* that can affect mucosal or cutaneous surfaces. It can manifest via buccal mucosa, associated with a skin lesion or as a secondary effect. Over the last 20 years, the number of cases of this disease is progressively increasing in Brazil. Therefore, the knowledge of this disease by health professionals is important in order to achieve a correct and early diagnosis, manly to prevent the deformities it may cause to the face. *Case presentation*. The aim of the present study was to report a case of mucocutaneous leishmaniasis with lesions on the palatine and pharyngeal mucosa in a patient with a previous report of rare lesions in the nasal mucosa and cartilage bone septal. *Conclusions*. We believe that the disclosure of such cases may be important for the correct and early diagnosis of these secondary injuries that may affect the oral mucosa.

## 1. Background

The American cutaneous leishmaniasis (ACL) is a chronic infectious disease, not contagious, caused by *Leishmania* protozoa belonging to the Trypanosomatidae family and can be present in two main ways: one is flagellated or promastigote, basically found in the digestive tract of the insect vector, and another is aflagellate or amastigote, found in the tissues of vertebrate hosts [1].

The most common transmission is through the insect bite that may belong to several species of sandflies, of different genera, depending on geographic location [1]. The CL can affect the skin, mucous membranes, and the mucocutaneous form is predominantly caused by *Leishmania braziliensis* [2].

Cutaneous leishmaniasis is a public health problem in 88 countries distributed on four continents (Americas, Europe, Africa, and Asia), with an annual registration of 1 to 1.5 million cases. It is considered by the World Health Organization (WHO), as one of the six most important infectious diseases due to its high detection coefficient and the capacity to produce deformities [3].

According to the WHO, most cases of cutaneous leishmaniasis occur in Afghanistan, Algeria, Brazil, Colombia, the Islamic Republic of Iran, Pakistan, Peru, Saudi Arabia, and the Syrian Arab Republic. Almost 90% of mucocutaneous leishmaniasis cases occur in Bolivia, Brazil, and Peru [4].

The epidemiology of mucocutaneous leishmaniasis in the Americas region is complex, with variations in transmission cycles, hosts, vectors, clinical manifestations, and response to treatment, due to the presence of multiple *Leishmania* species in the same geographic region [4].

It is a zoonotic disease in clear geographic expansion in Brazil, being one of the most important skin infections, not only due to the incidence, but mainly due to the therapeutic difficulties, deformities, and sequels that it may result [1]. It is particularly important in South America for presenting aspects of chronicity and latency and for developing metastases that lead to disfiguring clinical conditions. These injuries may result from a recurrent infection, whose origin could be a reactivation of a primary infection after a long period of latency or reinfection [5].

Primary skin lesions occur at the site of the sandfly bite, especially the lips and nose. The mouth can be involved by direct extension from the cutaneous lesion [2].

Recurrent leishmaniasis, which appears as a result of reactivation, has received more attention, not only due to the involvement of mucosal lesions, more difficult to treat, but also because they appear as a result of immunodeficiency states [5, 6].

The case reported refers to a female patient with lesions on the hard palate, soft palate, and pharynx, in addition to presenting nasal septum sequelae of a possible primary infection.

## 2. Case Presentation

A 52-year-old farmer, L. M. B., woman, resident in a rural area, attended the Oral Medicine Service, referred by a head and neck surgeon, for evaluation of a granulomatous lesion on the palate and complaining of hoarseness, which she believed to be a sequela of a flu occurred three months earlier. According to the doctor's report, the patient presented extensive oral lesion, having undergone a laryngoscopy and biopsy, with a histopathological result of pseudoepitheliomatous hyperplasia and an active chronic inflammatory process, suggesting a fungus investigation, the reason why the doctor referred her to our service.

During the health questionnaire, the patient did not reveal any systemic alteration as well as use of medications chronically. The patient reported having worked in the sugarcane fields for many years and had history of crusting and nasal bleeding, started about 4 years earlier. Physical examination showed nasal constipation, issuing nasal voice, and was mouth breather. The patient also reported to had been smoking for 6 months and the use of upper and lower total prosthesis.

**Figure 1:**
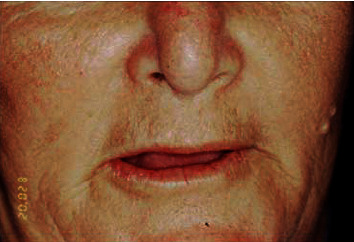
Defect in the nasal wing.

**Figure 2:**
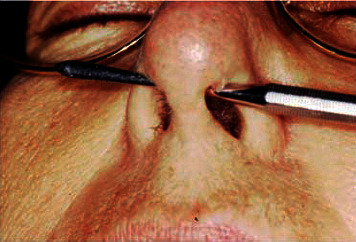
Nasal septum perforation.

Extrabuccal clinical examination revealed a lowering of the right side of the nasal wing and nasal septum perforation (Figures 1 and 2).

**Figure 3:**
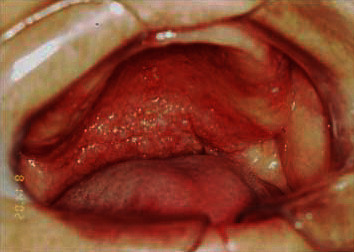
Granulomatous lesion on the palate.

**Figure 4:**
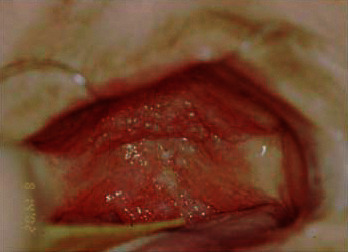
Lesion extending to the pharynx.

Intrabuccal examination revealed granulomatous lesion involving the entire palate and uvula, extending to the pharynx bilaterally with erythematous appearance and presence of scratchable white plaques distributed in some regions (Figures 3 and 4). The patient reported continuous use of the prosthesis.

Diagnostic hypotheses of mucosal leishmaniasis and paracoccidioidomycosis associated with candidosis were formulated. Mouth rinse using nystatin oral solution, hand hygiene instructions, and night removal of the prosthesis were prescribed. In posterior return, the hemogram and chest X-ray previously requested by the doctor were evaluated. The hemogram showed microcytosis and moderate hypochromia with basophilic dotting and discreet polychromatophilia. Chest X-ray analysis showed dense and congestive yarns. Radiographic examination of the bones of the nose, which demonstrated integrity of the nasal septum, was also requested.

Serology and mycological exams were requested. Direct mycological was negative. On clinical examination, no changes were noticed in the appearance of lesions after use of antifungal mouthwashes.

In view of the negative result of the direct mycological examination, sputum examination and the Montenegro intradermal test (MIT) for leishmaniasis were requested. In the same visit, incisional biopsy of the buccal lesion was also performed.

**Figure 5:**
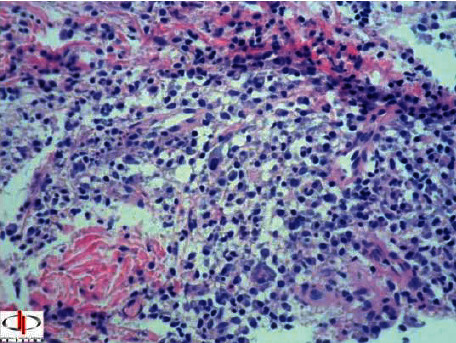
Result of the histopathological examination conducted by the Laboratory of Pathological Anatomy, Dr. Paino: ulcerative inflammatory process with lymph-plasmacytic predominance and possible leishmaniasis with etiological agent not demonstrable by the method of hematoxylin and eosin or giemsa (hyperallergic reaction?). A close clinical-epidemiological correlation is necessary, as well as with Montenegro intradermal reaction. Note: there is no evidence of malignancy.

After 7 days, the patient returned with the result of the histopathological examination (Figure 5), which revealed signs compatible with leishmaniasis, but without demonstration of the etiological agent.

**Figure 6:**
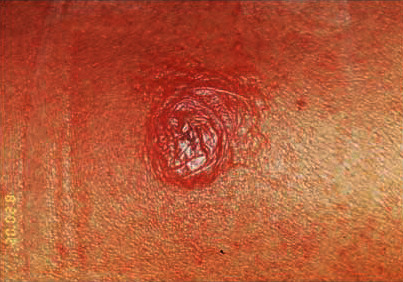
Positive Montenegro reaction (exacerbated).

The MIT test was positive with an exacerbated reaction (bubble formation) (Figure 6).

Once the definitive diagnosis of mucosal leishmaniasis was formulated, the patient was referred to the Araraquara Health Service (SESA) for treatment. At the same time, Rx of nasal bone was requested, which demonstrated integrity of the nasal bone septum.

Patient treatment consisted of daily administration of 3 ampoules of 5 ml of glucantime 425 mg, diluted in 250 ml of saline solution, administered intravenously for about 2 hours in a hospital for 30 days.

**Figure 7:**
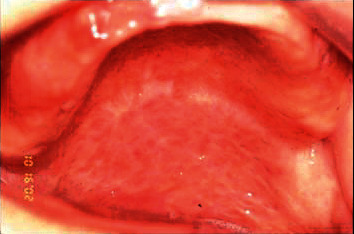
Clinical appearance after treatment with glucantime.

**Figure 8:**
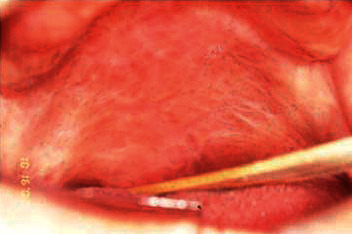
Clinical appearance after treatment with glucantime.

In a follow-up visit to our service, after completing the treatment, the patient reported stomach pain in the first 2 days of treatment; arthralgia and swelling of the joints of hands and legs during the course of treatment were also reported. Intraoral clinical examination was conducted where complete regression of the lesion was observed, leaving only cicatricial aspect (Figures 7 and 8).

The patient reported nasal decongestion and improved breathing. She was then referred to an otolaryngologist for evaluation of the nasal and laryngeal region, since the patient showed improvement in hoarseness. At the moment, the patient is followed up by this service, showing no other manifestation of the disease.

## 3. Discussion and Conclusions

Leishmaniasis is fundamentally a dermatozoonosis of wild animals, which can reach the man by contact to zoonotic outbreaks [1]. This parasitic infection is caused by intracellular organisms, found in tropical climates [7]. Its transmission occurs in a vector way through the female sandflies *Lutzomyia* and *Psychodopygus* [8]. This disease is endemic in 98 countries and affects more than 12 million people worldwide [9].

The largest number of patients affected by the disease is young male adults, who perform risky activities, mining, logging, and extractive activities, especially in the north and center-west of Brazil. There are also cases of leishmaniasis in other regions of the country, in old rural settlements, not associated with the clearing of the forests. In this pattern, dogs, horses, and rodents seem to play an important role as a causative agent reservoir, the *Leishmania* spp., and therefore the profile of the patients present change, reaching people of both gender and age groups [1, 10].

Cutaneous leishmaniasis has an incidence of approximately 1.2 million cases per year [3, 9]. It affects the skin and mucous membranes and is characterized by the presence of a well-delimited ulcer with raised borders [8]. This group of diseases that affects mainly the skin is called cutaneous leishmaniasis (CL) and is according to the World Health Organization, among the six infectious and parasitic diseases of major importance in the American continent, thus being an important public health problem due to its magnitude, transcendence, and low vulnerability to control measures [1, 5].

Some of the most common species of the genus *Leishmania* include *L*. *braziliensis*, *L*. *mexicana*, and *L*. *donovani*. This classification is based on clinical and epidemiological characteristics supported by biological, biochemical, and molecular aspects [5].

According to the Brazilian Ministry of Health (BMH), 30,000 new cases are diagnosed every year in Brazil [11]. The disease can be acquired through the bite of the insect vector (phlebotomine), when infected females inject the promastigote form of the organism. The incubation time has on average one month [1, 10, 12].

The maintenance of the infection in the host occurs as a consequence of the rupture of highly infected cells, when free amastigotes are phagocytosed by macrophages that reach the inflammatory focus or simply by macrophages division already colonized by the protozoan [1, 5].

The mucocutaneous leishmaniasis is almost always secondary to skin lesions, generally appear months or years after the resolution of lesions on the skin [1, 10, 13]. However, when it does not identify the gateway, it is assumed that the lesions originate in a subclinical infection [1, 6]. Despite the recurrence has the ability to affect any region of the digestive tract, there seems to be a predilection for the nose, leading to the appearance of a septal granuloma resulting in perforation of the nasal septum [13–15]. Therefore, the most common complaints in these cases are nasal obstruction, epistaxis, rhinorrhea, crusts, sore throat, hoarseness, coughing and ulcerations granulomatous in the oral mucosa [1], dysphonia, glottis edema, and drooling [10]. Colombo et al. [15], in 1992, published a comparative study of paracoccidioidomycosis and mucocutaneous leishmaniasis, which reported that chronic nasal obstruction was the complaint that led all leishmaniasis patients to seek medical assistance. We can see that in the case presented by us in this work, this same complaint was present, including causing too much trouble for our patient.

The buccal mucosa, the lesions mainly involve the posterior portion of the hard palate and the soft palate, with isolated lesions of the hard palate, is rare. These lesions are irregular, with granulomatous called “cobble street”, and in some cases, the uvula may be destroyed [6, 10, 14]. There may be partial or total destruction of the nasal pyramid with the fall of the tip of the nose for the destruction of the septum and subsepto, producing the so-called “bulldog face”.

The presence of one or more atrophic scars on the skin or skin ulcer history with prolonged course, associated with the aforementioned complaints, reinforce the clinical diagnosis of mucocutaneous leishmaniasis, but not always the absence of scars must set aside clinical suspicion of mucosal involvement by leishmaniasis, when the intraoral clinical picture is suggestive of the disease [1, 6]. In our case, it was observed scar on the skin of the nose and nasal septum perforation, with a prior history of ulcer in the region.

It should be considered in the differential diagnosis of mucosal lesions suggestive of leishmaniasis, paracoccidioidomycosis, the lepromatous leprosy, tertiary syphilis, the average facial granuloma, neoplasia, and histoplasmosis [1, 6]. São Thiago et al. [16], in 1998, published a case of histoplasmosis in the hard palate simulating a lesion caused by *Leishmania* and when the case of the patient described here was referred to us by the doctor, a search for fungi (*P*. *brasiliensis and H*. *capsulatum*) was requested.

The diagnosis of mucocutaneous leishmaniasis may be based on the disclosure of the parasite in the tissue and/or immunological tests. This disclosure can be carried out by direct (scarification impression by apposition) or indirect (histopathology, culture, or inoculation into laboratory animals) examination. The histopathology of biopsied lesions may be characteristic and suggestive for leishmaniasis but is rarely sufficient to make the diagnosis if the amastigotes are not identified in histopathological part [10, 12]. The Brazilian experience overall reveals not to be high sensitivity to this method because the success in the detection of parasites is inversely proportional to the evolution of injury time, with rare disclosure after a year of skin disease. Direct examination is the procedure of choice because it is faster, lower cost, and easily running [1, 10]. The immune diagnosis can be made by the Montenegro test, the enzyme immunoassays (ELISA), and indirect immunofluorescence. The Montenegro intradermal reaction translates the allergic response of late cellular hypersensitivity, and it is performed by intradermal inoculation of the antigen on the anterior aspect of the left forearm of healthy skin, 2 to 3 cm below the antecubital fold. The reading should be taken after 48 to 72 hours, and the induration is considered positive when the result is equal or greater than 5 mm. It is a highly predictive test due to its sensitivity, being positive in more than 90% of cases of leishmaniasis. In mucosal lesions, the positive Montenegro test is more severe and can occur even in ulceration and necrosis site, and in this case, a heightened reaction with blistering on site was observed. Indirect immunofluorescence and enzyme immunoassays express levels of circulating antibodies being made only in specialized referral centers. The positivity of these tests is associated with the time course of the disease and is more frequent in cases of mucous involvement [1, 10]. These tests are also used in the prognostic evaluation of treated mucosal lesions [6]. Most modern diagnostic methods include polymerase chain reaction (PCR) and in situ hybridization, and these techniques have high sensitivity and are able to detect and identify *Leishmania* species very quickly; however, they are expensive tests and require trained personal training, limiting their utilizations [10]. An important feature of mucosal injury is the latency because the parasite can remain for decades in the mucosa before starting the granuloma and the predisposing factors for this activation is not yet clear but may include a local trauma or immunosuppressive host conditions [6]. Immunosuppression by various causes, including infection by human immunodeficiency virus (HIV), resulting in increased susceptibility to leishmaniasis, often presents an atypical clinical course and a poor response to treatment [14]. Recently, several cases reported in the literature have associated their appearance with HIV infection [14, 17], including Chaudhry et al. [14] in 1999, who reported a case where the injury mucosa by leishmaniasis was the first sign of HIV infection.

The degree of deformity caused by leishmaniasis is highly variable, appearing sometimes as a simple perforation of the nasal septum to varying degrees of destruction of the center of the face. Secondary infections can be installed, requiring treatment with antibiotics, especially to prevent thrombosis of cavernous sinus [6]. The treatment of choice in cases of mucocutaneous leishmaniasis is the use of pentavalent antimony (glucantime), and some authors report that the mucous form responds poorly to treatment with these drugs and, despite the recommendation of its use by the World Health Organization (WHO), believe that their choice as the first choice is contradictory [12].

The recommended dose for mucosal lesions is 20 mg/Sbv/kg/day for 30 consecutive days, preferably in a hospital environment. The applications must be parenterally, intramuscularly, or intravenously, with advice to rest after application. There may be an exacerbation of clinical status at the beginning of the treatment leading to edema and acute respiratory failure. Thus, it is advisable that the medication is administered by a specialized team, under hospitalization and the possibility of emergency tracheotomy if necessary. Side effects can occur, such as arthralgia, myalgia, loss of appetite, nausea, vomiting, abdominal pain, fever, weakness, headache, insomnia, palpitations, and acute renal failure, among others [15].

Weekly electrocardiographic monitoring and evaluation of renal function should be performed, especially in patients over 50 years, and there is no complete healing after 12 weeks of complete treatment; the scheme will be repeated only once [1, 5]. Relapses can occur and are difficult to treat when it established, and there is no satisfactory response to treatment by pentavalent antimony. Drugs of second choice are amphotericin B and pentamidine [1, 6, 10, 13, 18]. In 1986, Magalhães et al. [19] published a study that showed no significant difference between the responses to treatment with glucantime and amphotericin B in 162 patients.

The cure criterion is the clinical regression of all signs and symptoms, evidenced by an otolaryngological exam within three months after completion of the treatment regimen [1].

It is recommended a monthly monitoring of the patient until the complete cure of injuries and annual indefinitely because of the potential for relapse after treatment with antimony [6].

The fight against the insect vector and disease control in host animals, treating the source of human infection, individual health protection, and preventive vaccines are the most important actions for the prophylaxis of leishmaniasis [10]. Vaccination as means of disposing of leishmaniasis has been studied, and much has been advanced in this research [5, 12].

The literature is consistent in saying that there is need for more accurate research on this disease, since the influx of immigrants from Latin America, and the permanence of the armed forces in endemic areas and the involvement of immunocompromising patients have increased the frequency and severity of injuries by leishmaniasis. The disease could become endemic in areas previously unaffected. We also believe that the disclosure of such cases may be important for the correct and early diagnosis of these secondary injuries that may affect the oral mucosa.

## Abbreviations

ACL: American cutaneous leishmaniasis (ACL)
BMH: Brazilian Ministry of Health (BMH)
HIV: Human immunodeficiency virus (HIV)
MIT: Montenegro intradermal test (MIT)
PCR: Polymerase chain reaction (PCR)
SESA: Araraquara Health Service (SESA)
WHO: World Health Organization (WHO).
